# Efficacy of Tuina in patients with chronic low back pain: study protocol for a randomized controlled trial

**DOI:** 10.1186/s13063-020-4198-2

**Published:** 2020-03-17

**Authors:** Shuaipan Zhang, Lingjun Kong, Qingguang Zhu, Zhiwei Wu, Jianhua Li, Min Fang, Wuquan Sun, Yanbin Cheng, Shanda Xu, Guangxin Guo, Xin Zhou, Zhizhen Lv

**Affiliations:** 1 Yueyang Hospital of Integrated Traditional Chinese and Western Medicine, Shanghai University of Traditional Chinese Medicine, Shanghai, 200437 China; 2 Institute of Tuina, Shanghai Institute of Traditional Chinese Medicine, Shanghai, 200437 China; 3 School of Acupuncture-Moxibustion and Tuina, Shanghai University of Traditional Chinese Medicine, Shanghai, 201203 China

**Keywords:** Low back pain, Efficacy, Randomized controlled trial, Tuina, Health care education

## Abstract

**Background:**

Low back pain is a common reason for medical care and carries a heavy social burden. The efficacy of Tuina or health care education for low back pain has been evaluated in previous systematic reviews. However, there is no evidence to support the superiority of one form of treatment over another. The aim of this study is to compare the efficacy of Tuina with health care education in the management of low back pain.

**Methods/design:**

This study is a randomized controlled trial with parallel-group design including two groups: a Tuina group and a health care education group. A total of 160 eligible participants will be randomly assigned to the groups in a 1:1 ratio. The interventions of both groups will last for 20 min and be carried out twice each week for a period of 12 weeks. The primary outcome is the Oswestry Disability Index. The secondary outcomes include a visual analogue scale and the 36-item Short Form Health Survey. They will be assessed at baseline, at the end of the intervention every month, and during 6 months and 9 months of follow-up by repeated measures analysis of variance. The significance level is 5%. The safety of Tuina and health care education will be evaluated after each treatment session. This study will focus on the value of Tuina and health care education for low back pain and will highlight any differences in the efficacy of the treatments.

**Discussion:**

This study will evaluate the efficacy and safety of Tuina intervention for low back pain, which could provide reliable evidence for clinical decision making for patients with low back pain.

**Trial registration:**

Chinese Clinical Trial Registry, ChiCTR1900022656. Registered on 23 April 2019.

## Background

Low back pain is a common symptom in today’s society. It causes serious health and economic burdens. A study showed that the health cost of low back pain was increasing in every year, and the number of persons with disability due to low back pain increased by 54% between 1990 and 2015 [1]. In high-income countries, the highest point prevalence of low back pain has reached 32.9% [2]. According to the epidemiologic data of low back pain in Asia, it was defined as a health problem associated with functional limitations and financial burden [3, 4].

Low back pain can be attributed to excessive physical exertion or trauma, resulting in damage or degradation of the vertebrae, intervertebral discs, or spinal muscles. Now, we focus more on nonspecific low back pain, which typically can account for 90% of the patients [5]. The duration of pain persists for at least 3 months and must meet the International Classification of Diseases, 11th Revision (ICD­11), criteria for chronic primary pain [6]. Owing to the considerable pain and cost associated with chronic pain, some interventions must be applied to prevent its exacerbation [7].

A treatment option for individuals with low back pain is a risk stratification approach depending on screening tools such as the Orebro Musculoskeletal Pain Screening Questionnaire [8] and the STarTBack tool [9], which aim to limit progression to chronic low back pain. With a high risk, regardless of the potential harm with some pain medicines, opioids [10] and nonsteroidal anti-inflammatory drugs [11] will be used to control the condition when it does not respond to nonpharmacological treatments [12]. Surgery will also be used to relieve the compression of nerve roots caused by degenerative disc disease [13]. However, some guidelines have endorsed the cautious use of medication and surgery and take nonpharmacological and noninvasive treatments as a first-line treatment, including routine health education, exercise, psychotherapy, and physical therapies, owing to the risk of trauma and the cost [14–17]. A relatively new and promising approach in the health care education of low back pain has focused on the neurobiology and neurophysiology of pain, which attempts to help the patients understand that pain and tissue injury are different constructs [18, 19]. In addition, education is composed of the factors that affect the occurrence of the disease, including weight, occupation, and exercise style; explanation of the natural development process and harm of the disease; and explanation of the planning and prognosis of disease treatment. For patients with chronic low back pain, several studies have shown favorable outcomes following health care education in patients with chronic pain in terms of pain relief and improved physical performance [20–22]. It can also reduce emotional distress and subsequent health care use [23, 24].

Additionally, some systematic reviews showed a moderate level of evidence for the clinical efficacy of manual therapy, massage, and spinal manipulations in patients with low back pain [25–27]. Tuina, one of the key noninvasive therapies of traditional Chinese medicine, is a Chinese manual manipulation. It has been used for thousands of years in China. Some studies showed the potential effect of Tuina for chronic neck pain [28], but more clinical trials are required to provide evidence of Tuina for low back pain. Health care education has also shown promise in previous reviews to improve pain and disability in chronic low back pain [29, 30]. Therefore, this study was designed to compare the efficacy of Tuina with health care education for patients with low back pain.

## Methods/design

### Study design

**Fig. 1:**
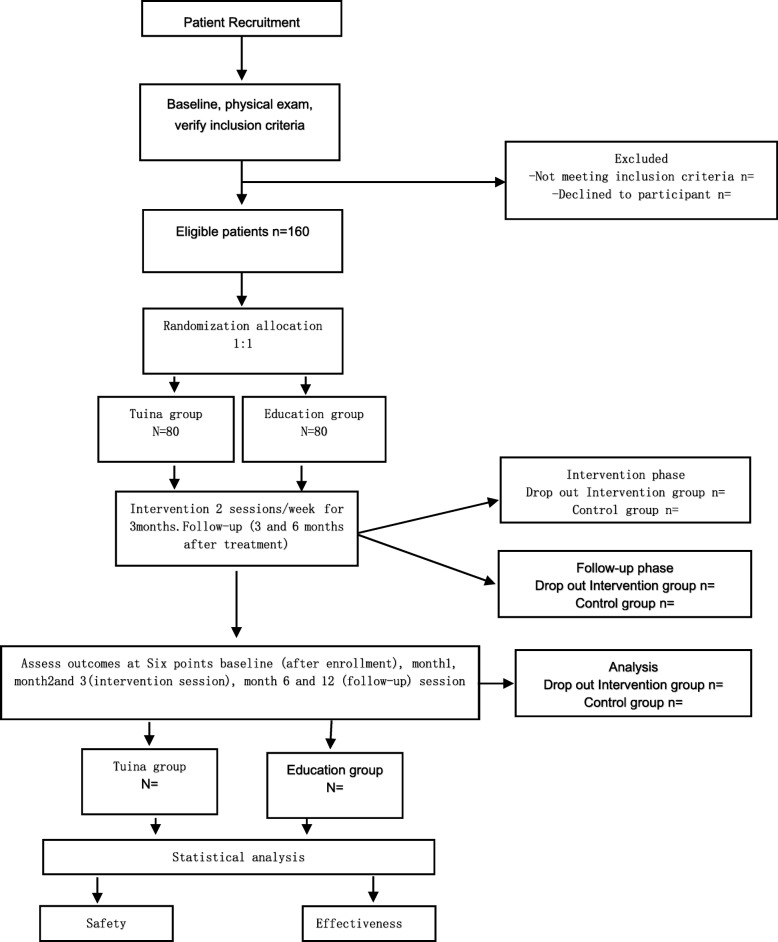
Flowchart of the study. A total of 180 participants will be randomized to the two groups. The interventions will last for 20 min and will be conducted two times per week for 12 weeks. The study period will consist of the baseline, 3 months of treatment, after treatment, and 3- and 6-month follow-up. *ODI* Oswestry Disability Index, *SF-36* 36-Item Short Form Health Survey, *VAS* Visual analogue scale

**Fig. 2:**
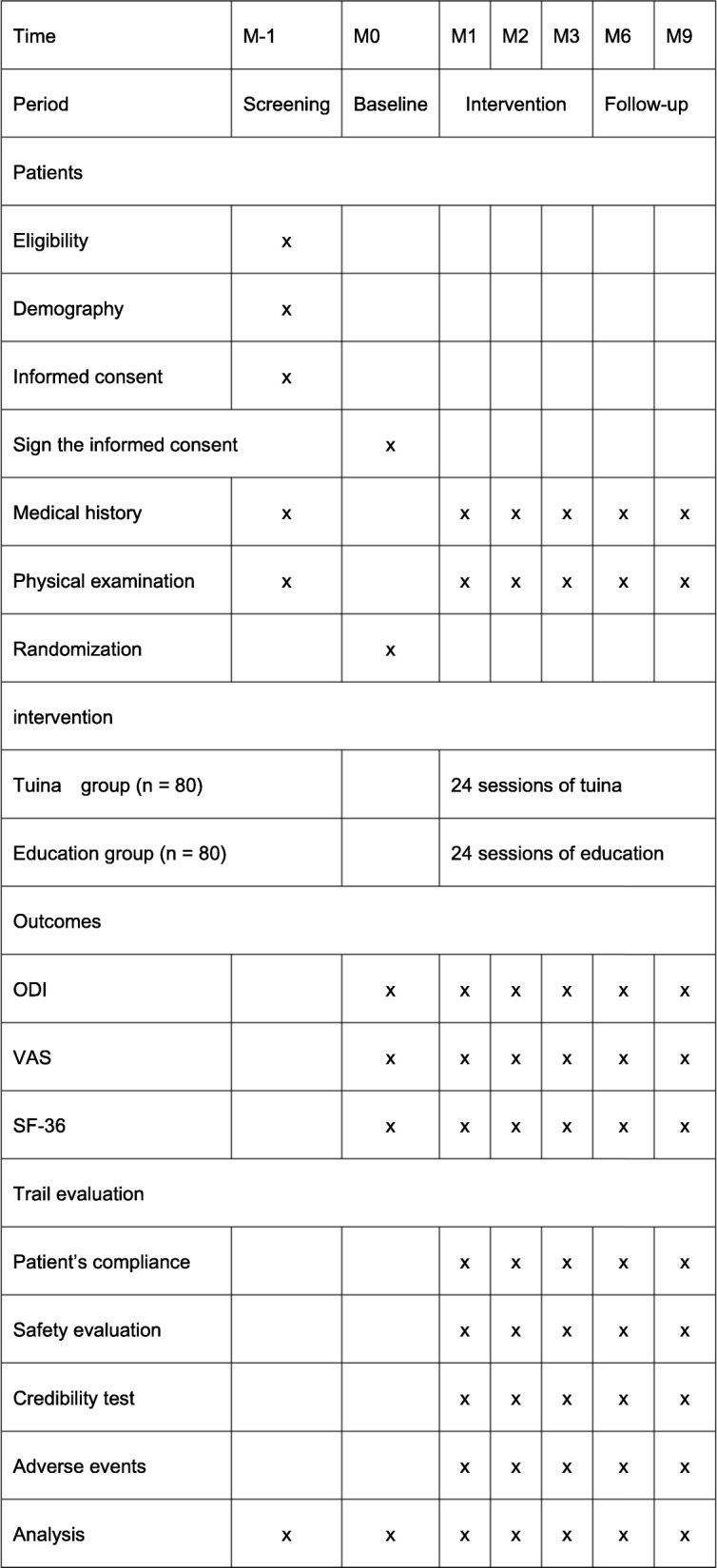
Study schedule

This study is a single-center, assessor- and analyst-blinded randomized controlled trial conducted in Shanghai, China, at Yueyang Hospital of Integrated Traditional Chinese and Western Medicine affiliated with Shanghai University of Traditional Chinese Medicine. In total, 160 patients will be recruited and randomly assigned to a Tuina group and a health care education group in a 1:1 ratio. This trial protocol was approved by the Ethics Committee of Yueyang Hospital of Integrated Traditional Chinese and Western Medicine affiliated with Shanghai University of Traditional Chinese Medicine (project number 2019-035). It is registered with the Chinese Clinical Trial Registry (ChiCTR1900022656). Written informed consent will be provided by all patients before the screening. The interventions will be given twice per week for 3 months. We will ask some researchers who are blinded to assignment to accomplish the outcome assignment and statistical analyses independently. The trial flowchart and study design are shown in Figs. 1 and 2, respectively.

### Participant recruitment

Patients with chronic low back pain consistent with the disease definition in the ICD-11 will be recruited. The participants are outpatients and inpatients in Tuina departments of Yueyang Hospital of Integrated Traditional Chinese and Western Medicine affiliated with Shanghai University of Traditional Chinese Medicine. We will also seek potential patients through posters, Internet advertisements, and the official microblog and WeChat platforms. All participants are aged 18 to 65 years. We define 10 years as an interval, and the number of participants in each interval is almost equal.

### Inclusion criteria

The inclusion criteria are as follows:

1. Duration of low back pain as the main symptom for at least 3 months
2. Pain lasting more 20 min per time and at least once per month
3. Aged between 18 and 65 years, male or female
4. Volunteer to take part in the study and sign the informed consent form
5. Promise not to receive other related therapy during the period of treatment
6. Capable of complying with the intervention and follow-up assessment

### Exclusion criteria

The exclusion criteria are as follows:

1. Chronic low back pain caused by local disease (e.g., lumbar fracture, lumbar tumor, lumbar tuberculosis, lumbar spine surgery or trauma)
2. Sciatica, myelopathy, displacement, or radiculopathy due to lumbar intervertebral disc disorders or spondylolisthesis
3. Severe primary disease such as cardiovascular, lung, kidney, and hematopoietic disease
4. Ongoing treatment within 5 days prior to inclusion
5. Mental disorder
6. Pregnant woman
7. Patients not suitable for nuclear magnetic resonance examination

### Dropout and suspension criteria

Patients have the right to withdraw for any reason during the study period according to the Patient Management Protection Rules. The following conditions are considered as withdrawal criteria: (1) patient does not implement the protocol treatment on schedule; (2) participation in other treatments during the trial; (3) intolerable adverse events; and (4) lost to follow-up. In addition, if severe poor efficacy or adverse reactions occur during the trial, the trial will be forcibly suspended immediately.

### Randomization

The randomization sequence will be generated by a random number generator (IBM SPSS Statistics version 21.0 software; IBM Corp., Armonk, NY, USA), which will be sent to a therapist with an opaque envelope numbered sequentially. The therapist will sequentially open random-allocation envelopes and allocate the participants accordingly. Eligible patients will be randomized to the Tuina group or the health care education group, with 80 patients in each group.

### Blinding

Participants and therapists will not be blinded to treatment allocation, owing to the limitation of the therapies. Interventionists will be masked to the participants’ outcome measurements. The evaluators, data managers, and statisticians will be blinded to the group allocation in the outcome evaluation procedure and data analysis for the sake of reducing the risk of bias.

### Interventions

The participants will receive a total of 24 treatments in 3 months. The therapists must have 10 years of experience with Tuina treatment or be skillful in health care education for the participants. In addition, they are required to have passed a clinical test to make sure that the consistency of the trial is administered. It should be emphasized that some other treatments for low back pain during the trial will be forbidden, which includes medication therapy, surgery, drug injections, acupuncture, moxibustion, and physical therapy. If the subjects receive any other treatment, it will be recorded the changeable each time he arrives.

### Tuina group

The tendons and bones theory of traditional Chinese medicine summarizes the physiology and pathology of physical movement. The relationship between tendons and bones in the human body is inseparable. During body movement, the muscles will contract to produce force and transfer it to the bone with the help of ligaments or tendons. At the same time, the bone can efficiently process the force to different parts of ligaments and tendons, resulting in a coordinated motion pattern. Coordination of tendons and bones is the main basis for maintaining the dynamic balance of the spine and joints of the body. Tuina of “restrict bone and harmonize joints” intends to restore the harmonious relationship between tendons and bones by manipulation of the low back with the therapist’s hands. The specific process is outlined in the subsections below.

### Step 1: local manipulation

First, the patients are in a prone position. The doctor stands on the side of the patient with the method of rolling the Bladder meridians on both sides of the spine, which is applied for 3 to 5 min. The waist will be focused on to manipulate preferentially and then using the same technique on the patient’s buttocks and lower limbs at the same time. It plays the role of relaxing sinews and activating collaterals with the theory of traditional Chinese medicine. Second, the physician will perform the manipulation on the patient’s waist and the posterolateral side of the lower limbs by pressing, kneading, plucking, and so forth for 5 to 7 min, which can relieve the cramping of the muscles. The doctors press the acupoints, including Mingmen (DU04), Shenshu (BL23), Yaoyangguan (DU03), Huantiao (GB30), Weizhong (BL40), Chengshan (BL57), and Ashi points, with the elbow or the thumb to achieve Deqi sensation, which is commonly regarded as an indicator of manipulation efficacy [31–34]. Last, scrubbing manipulation will be used to dilate the heat with the palms. In conclusion, the above content refers to therapeutic manipulation for sinew injury.

### Step 2: lumbar structural rectification

Pulling manipulation on the lumbar vertebrae will be used to regulate the pathological condition of the disorder of the facet joint after finishing the relaxed manipulation [35]. First, the patient is required to face the doctor on the side of the bed in a lateral position. The lower limb in the upper part is on the knee, and the other lower limb is naturally straight. The physician stands on the ventral side of the patient, with elbow or hand against the front of his shoulder and the other elbow against the buttocks. The doctor will coordinate the strength with two hands or elbows, which begins with a small amount of rotation of the waist to relax the waist of the patient. Then, therapists gradually increase the angle of rotation of the patient’s waist with relative force until the maximum extent. It usually ends up with the sound of a “click,” which is without special pursuit during the clinical treatment. The same technique is applied to the contralateral lumbar vertebrae.

### Health care education group

The education intervention will be given to the participants with two sessions per week. The first health education session is a group meeting including all of the participants in the control group using a PowerPoint presentation, which will last 30–60 min. An educational booklet and a video will be given to the patients to do the home-based online e-learning module, containing the same content provided in the PowerPoint presentation. The second session will be conducted as one-to-one communication focused on personal needs to guide patients to implement the key education in their lives. This intervention will be maintained by an experienced physical therapist for 12 weeks. The program will include the following two broad components: (1) introduce key concepts of pain biology and (2) present specification of recovery.

Each patient will be given an introduction about the underlying mechanisms, predisposing factors, and prognosis of low back pain. Pain will be explained as the conscious part of the response, which can be influenced by many factors as a protective output. Interventionists will formulate specification of daily routines for patients, for example: keep the back warm; keep a healthy diet and prevent obesity; maintain beneficial posture and abandon bad habitual posture, keeping a normal physiological curvature of the spine; avoid overwork, intense activity, or lifting heavy weights; maintain proper exercise; and maintain a good mental state and be prepared to endure chronic pain patiently for a long time.

### Outcome measurements

The outcome will be measured by three self-report questionnaires, which can reflect the lumbar dysfunction, pain, and quality of life. Six time points will be used to assess outcomes, including baseline (after enrollment); months 1, 2, and 3 (intervention session); and months 6 and 9 (follow-up sessions).

### Primary outcome measurement

#### Oswestry Disability Index

The primary outcome is the subject’s perceived disability, which is assessed by the modified Oswestry Disability Index (ODI) [36]. The ODI consists of ten items, including pain intensity, personal care, lifting, walking, sitting, standing, sleep, sex life, social life, and traveling. According to the extent of disability, each item is scored from 0 to 5. The total score is reported as a percentage, which is actual score/50 (maximum possible score) × 100%. The ODI results will be assessed using a repeated longitudinal analysis.

### Secondary outcome measurement

#### Visual analogue scale

A visual analogue scale (VAS) is a 10-point scale selected to quantitatively measure the level of low back pain during the study, for which 0 means “none of pain” while 10 represent “the unbearable pain [37]”. It has been proved to have validity and reliability for outcome measurement. We will assess the VAS with a repeated longitudinal analysis.

#### Short-Form Health Survey

The association between health-related quality of life and various factors will be assessed by using the 36-item Short Form Health Survey (SF-36), which includes 36 questions divided into 9 areas. It consists of vitality (4 items), physical functioning (10 items), bodily pain (2 items), general health (5 items), physical role (4 items), emotional role (3 items), social functioning (3 items), mental health (5 items), and health transition (5 items). For each field, scores range from 0 to 100, with higher scores reflecting a better quality of life. A repeated longitudinal analysis will also be used to evaluate the SF-36.

#### Safety evaluation

Adverse events refer to the unexpected responses that occur during or after treatment, which can lead to a hospitalization or even threaten life. The trial should be suspended and immediate process is indispensable whenever we encounter adverse events. Also, we should record the details in the case report forms (CRFs). The efficacy and safety of the intervention will be evaluated with 6 months of unsupervised follow-up, during which the subjects should only be received routine lumbar care. At month 6 and 9, participants will be contacted with the phone for their physical condition of low back pain currently. In addition, evaluators can be reformed 13 with the clinical symptoms and AEs whenever the patients meet something important.

#### Data collection and monitoring

The screeners will collect data on the baseline characteristics when the patients are recruited, and CRFs will be completed with the outcome measurement by the assessor. This consists of questionnaire-based assessment of treatment effects, lumbar physiological function, adverse events, and safety evaluations. Then two data administrators, who are beyond the research team and blinded to group allocation, will independently receive the completed CRFs and enter them into an Excel database (Microsoft, Redmond, WA, USA). They are required to have completed rigorous training for the data monitoring. Then they will entry the real-time data in the Chinese Clinical Trial Registration Center, in which the electronic data management system will be used to track and monitor the test data in real-time in the Department of Science and Technology in Yueyang Hospital.

#### Statistical analyses

The intention-to-treat principle will be followed in the primary data analysis with IBM SPSS Statistics for Windows version 21.0 software by statisticians who are blinded to the group allocation. The baseline characteristics will be expressed with descriptive statistics for the two groups, which are reported as the mean ± standard deviation. A Kolmogorov-Smirnov test with Lilliefors correction will be used to analyze all quantitative variables to determine whether they follow a normal distribution. Parametric statistics (Tukey test) or nonparametric statistics (Wilcoxon rank-sum test) will be used for the within- and between-group analyses in accordance with the results of the homogeneity and normality analyses. When initial homogeneity and normality of data distribution are found, repeated measures analysis of variance (ANOVA) and ANOVA with Bonferroni *post hoc* statistics will be used to analyze within and between groups. The Friedman test and Kruskal-Wallis test will be used when initial homogeneity but not normality of data distribution is found. If the initial homogeneity is not found, a linear mixed model will be adjusted for the baseline value. Adverse events in each group will be documented as percentage for safety assessments using the chi-square test or Fisher’s exact test. The statistical significance is defined as *P* < 0.05, and the 95% confidence interval will be reported.

#### Sample size calculation

The rate of the expectation of efficacy in the Tuina group is 95% and in the health care education group is 85%, based on the literature. The sample size was calculated with a significance level of 0.05 and power of 0.80, which also considers a maximum dropout tolerance of 10%. Ultimately, 160 patients are needed for the trial, with 80 for each group.

#### Quality control

With the management of the steering committee, quality control will be conducted during the processing of the trial. Professional trial method and regular monitoring technique should be trained before the researchers participate in the trial, which can ensure the consistency of methods. The steering committee and ethics committee should be informed if the study protocol is modified or corrected.

## Discussion

A survey showed that low back pain has a high prevalence rate across the lifespan, which seriously affects quality of life. In addition, the medical cost is a leading contributor to disease burden wordwide [4, 38, 39]. Systematic reviews of risk factors for low back pain have suggested that the rising risk of back pain episodes is related to both physical and psychological risk factors [40]. It can be caused by prolonged standing and lifting heavy weights, an unhealthy lifestyle such as smoking and obesity, experiencing distress, and the expectations that pain indicates bodily harm or injury. So, targeted health education in primary care is necessary and has been shown to provide long-term reassurance for patients with low back pain [23].

Tuina therapy belongs to the key noninvasive therapies of traditional Chinese medicine, which is based on the theory of meridians and acupoints and modern anatomy. It takes advantage of the hands of the therapist to manipulate the surface of the patient, including relaxation techniques, such as pressing, kneading, and pushing, and some adjustment manipulations, such as pulling and shaking. The Huangdi Inner Classic Pain Theory states that “when your body feels pain, it means the balance in your body is disrupted.” Conversely, you will not feel any discomfort when your body’s qi is flowing smoothly through the meridians. Blood stagnation is caused by the stagnation of qi, which can lead to phlegm pattern as the process of the disease. The most obvious manifestation is characterized by stabbing pain in a fixed location, usually aggravated at night. Tuina can help the patient achieve a physiological balance of “blood and qi” by manipulation of “restrict bone and harmonize joints.” It has been found that patients with low back pain have a symptom that the muscle tension increases with local inflammatory stimulation and even continues to sputum in some biomechanical studies. Tuina is used to loosen muscle adhesions and significantly improves muscle strength of patients with low back pain. It can relieve the pathological fatigue state of the muscles, harmonize the coordinated balance function of the flexors and extensors, and finally restore a physiological balance of muscles and bones [41]. In previous studies, a rat model was established to demonstrate that Tuina could reduce pain sensitivity and peripheral nociceptive peripheral C-fiber activity in rats and could accelerate the repair peripheral nerve injury [42, 43].

However, the efficacy and safety of Tuina therapy requires further clinical proof. This trial is designed as a clinical randomized controlled trial to compare the efficacy of Tuina and health care education in patients with low back pain. The ODI, VAS, and SF-36 are being used as the outcome measurements, which can evaluate the efficacy of relieving pain and improve the quality of life with the interventions. This protocol intends to provide a more powerful evidence-based proof for Tuina therapy in patients with low back pain.

### Study limitations

There is an inevitable limitation that it is difficult to control the methodology of blinding during the physical intervention. In this study, Tuina and health care education are different forms of physical therapy, which cannot be blinded for the participants and therapists. Therefore, the blinding quality should be monitored to control the questionnaire measurement credibility, which is composed of the assessors, the administrators, and the data analysts.

## Trial status

This trial is still in the process of recruiting process. Participant recruitment started in June 2019 and will end in June 2020. This trial was registered in Chinese Clinical Trial Registry on 23 April 2019. The registration number is ChiCTR1900022656.

## Abbreviations

ANOVA: Analysis of variance
CRF: Case report form
ICD­11: International Classification of Diseases, 11th Revision
ODI: Oswestry Disability Index
SF-36: 36-Item Short Form Health Survey
VAS: Visual analogue scale
